# Web-based visualisation and analysis of 3D electron-microscopy data from EMDB and PDB^☆^

**DOI:** 10.1016/j.jsb.2013.09.021

**Published:** 2013-11

**Authors:** Ingvar Lagerstedt, William J. Moore, Ardan Patwardhan, Eduardo Sanz-García, Christoph Best, Jason R. Swedlow, Gerard J. Kleywegt

**Affiliations:** a Protein Data Bank in Europe, European Molecular Biology Laboratory, European Bioinformatics Institute, Wellcome Trust Genome Campus, Hinxton CB10 1SD, United Kingdom; b Centre for Gene Regulation and Expression, College of Life Sciences, University of Dundee, Dow Street, Dundee DD1 5EH, United Kingdom

**Keywords:** EMDB, Electron Microscopy Data Bank, EM VTC, Electron Microscopy Validation Task Force, FSC, Fourier Shell Correlation, OAV, Open Astex Viewer, OME, Open Microscopy Environment, OMERO, OME Remote Objects, PDB, Protein Data Bank, PDBe, Protein Data Bank in Europe, wwPDB, Worldwide Protein Data Bank, 3DEM, Three-Dimensional Electron Microscopy, Electron microscopy, Electron tomography, Visualisation, Macromolecular structure

## Abstract

The Protein Data Bank in Europe (PDBe) has developed web-based tools for the visualisation and analysis of 3D electron microscopy (3DEM) structures in the Electron Microscopy Data Bank (EMDB) and Protein Data Bank (PDB). The tools include: (1) a volume viewer for 3D visualisation of maps, tomograms and models, (2) a slice viewer for inspecting 2D slices of tomographic reconstructions, and (3) visual analysis pages to facilitate analysis and validation of maps, tomograms and models. These tools were designed to help non-experts and experts alike to get some insight into the content and assess the quality of 3DEM structures in EMDB and PDB without the need to install specialised software or to download large amounts of data from these archives. The technical challenges encountered in developing these tools, as well as the more general considerations when making archived data available to the user community through a web interface, are discussed.

## 1 Introduction

The Electron Microscopy Data Bank (EMDB; Tagari et al. (2002)) archive was established at the European Bioinformatics Institute (EBI) in 2002 as a means of making 3D macromolecular reconstructions derived from 3D electron microscopy (3DEM) experiments freely and publicly available. It has since then become the authoritative global archive for such data, comprising over 2000 released maps (October 2013) derived from a range of 3DEM techniques including single-particle reconstruction, helical reconstruction, tomography and electron crystallography. In 2012, over 400 new maps were released.

Since 2007, EMDB has been managed jointly by the EMDataBank partners (http://emdatabank.org/; Lawson et al., 2011): the Protein Data Bank in Europe (PDBe), the Research Collaboratory for Structural Bioinformatics (RCSB PDB), and the National Center for Macromolecular Imaging (NCMI). Nowadays, 3DEM maps and models can be deposited at PDBe, RCSB and the Protein Data Bank Japan (PDBj). The deposited 3DEM data is ultimately stored in the EMDB ftp archive at EBI and from there redistributed together with the PDB archive through the ftp sites of the Worldwide Protein Data Bank partners (wwPDB; http://wwpdb.org/; Berman et al., 2003). In addition, PDBe, RCSB and PDBj disseminate the 3DEM data in EMDB and PDB through their individual websites and services.

The mission of PDBe (http://pdbe.org/) is “Bringing structure to biology” and this underpins our ambition to become an integrated resource of structural and related biological information for use by the entire biomedical community (Velankar and Kleywegt, 2011; Velankar et al., 2010, 2011, 2012). As part of this effort, a number of web-based tools and services are being and have been developed that facilitate discovery and analysis of 3DEM data. Expert users benefit from a powerful search and browse service for EMDB data (EMSearch; http://pdbe.org/emsearch; EMBrowse; http://pdbe.org/embrowse) as well as a data-mining tool (EMStats; http://pdbe.org/emstats). This paper describes a set of web-based tools developed at PDBe that facilitate visualisation, analysis and validation of archived 3DEM data by expert and non-expert users alike. The tools include a 3D volume viewer for interactive visualisation of 3DEM maps and models, a slice viewer for inspecting 2D planes from tomographic reconstructions, and visual analysis pages that aid in the assessment of 3DEM data. All three tools work inside standard web browsers and therefore do not require users to explicitly download data or install special software. Here, we describe the main features of these tools, and discuss the technical challenges and limitations imposed by the browser environment.

Other efforts to visualise archived EM data include PDBj’s EM Navigator (http://pdbj.org/emnavi; Kinjo et al., 2012) and Pepper (Macias et al., 2007). EM Navigator is a web-based service for browsing EMDB data. One of its popular features is that it provides a movie for every entry where the structure is first rotated and then sliced. Pepper is a Java desktop application that combines 3D visualisation of overlaid maps and models with an interactive view of annotations of the molecules in the models, such as the model sequence, provided via the Distributed Annotation System (DAS; Dowell et al., 2001).

## 2 Volume viewer

**Fig.1:**
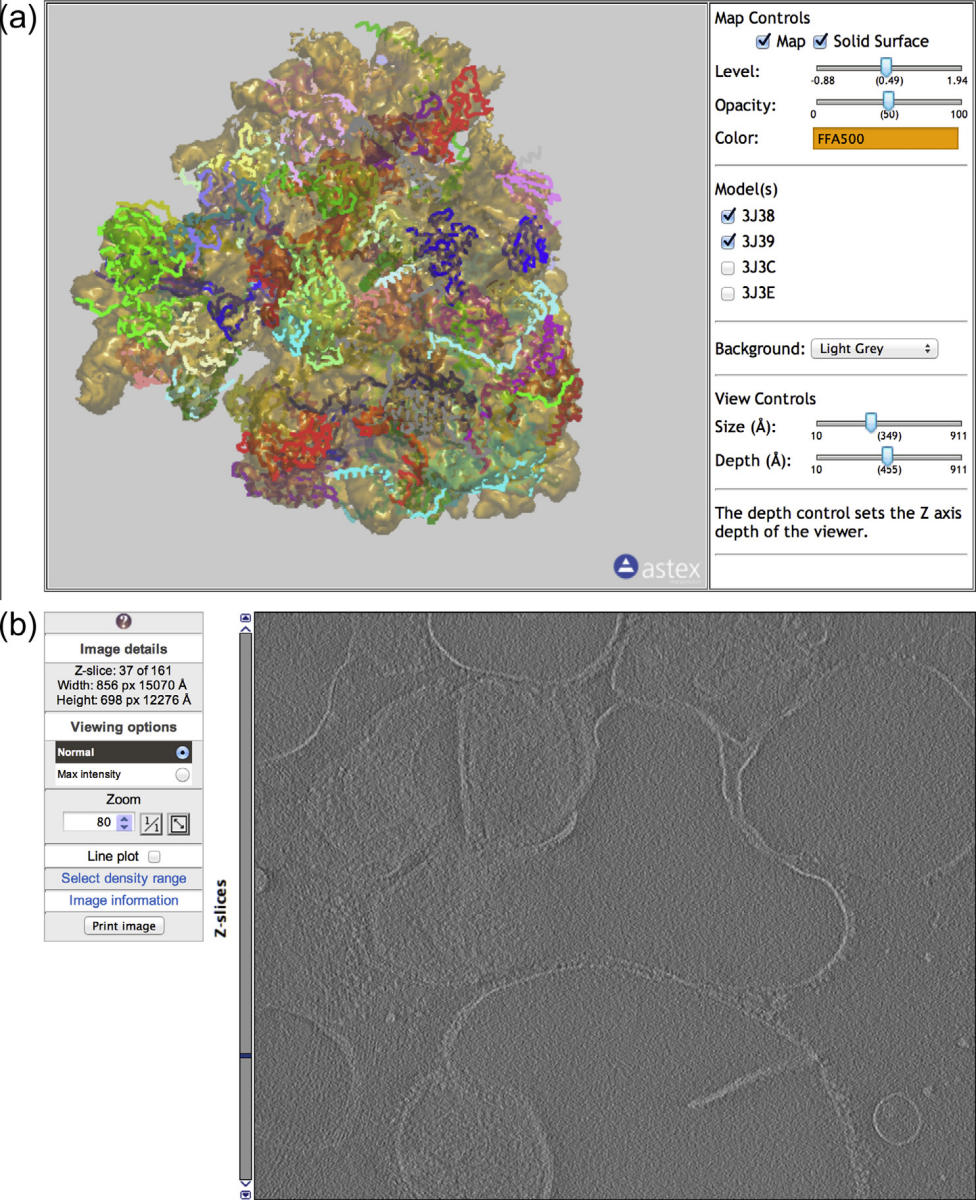
Interactive viewers for 3DEM data in EMDB and PDB. (a) Volume viewer showing EMD-5591 (80S *D. melanogaster* ribosome) and PDB entries 3j38, 3j39, 3j3c and 3j3e (Anger et al., 2013). The viewer contains the EM volume and a trace representation of the fitted models. (b) Slice viewer with EMD-1906 (tomogram of thylakoid membranes from spinach chloroplasts). The slice viewer only displays one plane at a time, which avoids the need to down-sample the map.

A user who wants to examine 3DEM data from EMDB and PDB typically needs to download maps from EMDB, models from PDB, and to examine these using specialised visualisation software such as Chimera (Pettersen et al., 2004) or PyMol (DeLano, 2002). This procedure can be time-consuming even for expert users and for this reason we have developed an interactive 3D volume viewer that allows for integrated visualisation of 3DEM maps and models inside a standard web browser. The viewer supports interactive surface rendering of 3D volumes of individual EMDB entries as well as overlays of fitted models from the PDB. The viewer can be accessed using URLs of the form http://pdbe.org/EMD-NNNN/volume, where “EMD-NNNN” is an EMDB accession number, for instance EMD-5591, see Fig. 1a.

The 3D visualisation engine of the viewer generates a surface rendering of a map at any desired contour level. It is a Java applet augmenting the Open Astex Viewer (OAV) applet (http://openastexviewer.net/; Hartshorn, 2002). The interface for interacting with the applet inside the browser relies on a combination of HTML and JavaScript. The main features of the viewer, including its ability to overlay related PDB models, and the technical challenges faced when visualising large and complex maps in the context of a browser are described below.

The volume viewer uses the OAV as a Java applet, and the memory at its disposal is therefore limited by the web browser. The upper limit for memory available to Java applets is typically 64–128 MB for the most popular browsers such as Firefox, Internet Explorer, Google Chrome, Safari and Opera. This limit can usually be increased, but the manner in which memory settings can be changed is not standardised between browsers, and users may feel uneasy making such changes themselves. With these concerns in mind, the aim was to limit memory usage to 128 MB for the vast majority of EMDB entries.

Maps are converted from the standard EMDB map-distribution format (http://pdbe.org/emschema) to Brix format (Jones et al., 1991) with 8-bit signal depth, *i.e.* one byte per voxel. The Brix format is similar to the DSN6 format used in X-ray crystallography since the 1970s, but with the header section written as text; it was developed by M. Kjeldgaard for use in the crystallographic model-building software O (Jones et al., 1991). Although electron-microscope detectors tend to have 10–12 bit signal depth (Saijo and Shiojiri, 2010), compressing the signal to 8 bits does not significantly change the visual appearance. OAV was modified to handle the 8-bit data representation internally, including in the existing marching-cubes algorithm that is used to generate surface representations (Lorensen and Cline, 1987).

Large EM volumes are down-sampled by trilinear interpolation, so that no dimension of a map exceeds 160 grid points. The memory footprint of the volume viewer after reducing data to 8-bit values and down-sampling is less than 128 MB, including ∼1.5 MB for the application code and up to 4 MB for the map (160 × 160 × 160 voxels maximum); the rest is available for the arrays required for 2D and 3D graphics objects. The cut-off value of 160 grid points is a compromise – for the majority of EM volumes, most structural details are retained while using a modest amount of memory. However, memory usage in a browser is cumulative and opening several instances of the volume viewer in the same browser may exhaust the browser’s available memory. If this happens one or more instances of the volume viewer applet need to be closed and the one that is actively used needs to be reloaded.

**Fig.2:**
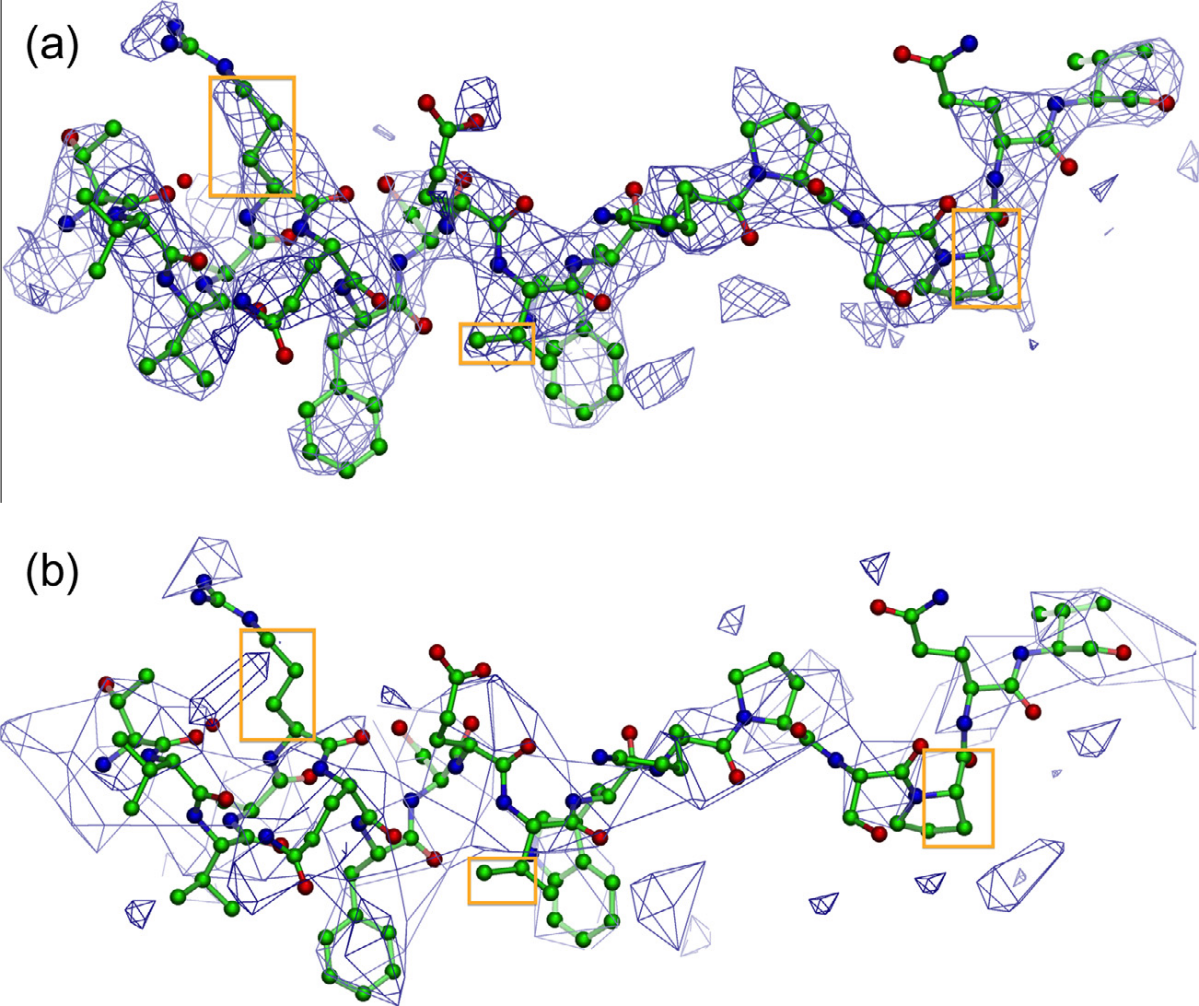
Effect of down-sampling. Comparing a deposited map with the down-sampled map used by the volume viewer. Most of the secondary structure detail remains, but side-chain density evident in the original map is lost in the down-sampled map. The 3.3 Å resolution map is of a helical reconstruction of Tobacco Mosaic Virus, EMD-5185 (Ge and Zhou, 2011) with fitted PDB model 3j06 (chain A, residue 41–58 shown here). (a) Detail of deposited map, 512 × 512 × 512 voxels. (b) Detail of map down-sampled to 160 × 160 × 160 voxels. The surface is rendered at a lower density level, 3.7, than the recommended level used for the deposited map, 6.42; this is done to give the volumes similar visual appearance.

The compression and down-sampling can lead to loss of detail in high-resolution maps: side-chain definition in the density may be reduced, but secondary-structure details tend to remain visible, see Fig. 2. Moreover, down-sampling to a grid spacing larger than half the map resolution is expected to lead to loss of detail. Finally, the volume viewer can only provide a meaningful 3D representation of the content in a map if all objects can be represented as a surface at a single contour level. For tomograms this is often not the case and the situation is further compounded by the loss of detail from bit-compression and down-sampling. Hence, we recommend use of the slice viewer (see below) for the visualisation of tomograms.

For some EMDB entries there are accompanying atomic models that have been deposited in the PDB. Visualising 3DEM volumes and atomic models together provides insight into how the various components of the sample are arranged and interact. For some entries, a complete atomic model is available, but for 2D-crystals, viruses, and helical entries usually only the asymmetric unit has been deposited, together with a set of transformations. The symmetry transformations (taken from the PDB entries, or defined by the planar group for 2D crystals) are used by the viewer to generate symmetry-related instances, according to the following rules:

• For viruses, generate a penton, *i.e.* copies around one of the 5-fold axes (for instance http://pdbe.org/EMD-5495/volume (Zhang et al., 2012)).
• For helical entries, 5 repeats are shown based on the associated symmetry operators for the model (for instance http://pdbe.org/EMD-5352/volume (Fujii et al., 2012)).
• For 2D crystals, show all copies inside the asymmetric unit (for instance http://pdbe.org/EMD-2219/volume (Abe et al., 2012)). The symmetry-related instances are translated to the unit cell of the EM volume. At least half of a symmetry-related instance must lie inside the EM surface for it to be displayed.

PDB models are converted to a simplified representation which is read by the viewer. This reduces the memory usage when multiple models have been fitted to the same map. The simplified representation is particularly important when displaying structures with high symmetry. For instance the memory needed to completely render 1, 5, and all 60 symmetry copies of PDB entry 3iyn (human adenovirus type 5 (Liu et al., 2010)), fitted into EMD-5172, is 33, 165, and 2001 MB, respectively. Using the simplified representation, this reduces to 0.9, 4.3, and 52 MB, respectively, thus making it feasible to show, and interactively manipulate, the entire virus assembly in a web-based viewer.

The simplified representation is based on an existing data structure in OAV, with extensions for annotations about model, chain, residue, and colour. Each residue is represented by a trace atom (CA for proteins, P for nucleotides, and the first atom of any other compounds) thereby allowing for the visualisation of secondary structure but not of side-chain detail. At present, the loss of side-chain detail only affects a handful of entries though this is likely to change as more and more high resolution structures are deposited.

The time it takes to load a map in the volume viewer depends on the user’s download speed. Most maps are 4 MB after down-sampling, the applet itself is just under 2 MB, and model files vary from 100 kB for some 2D crystals to 2 MB for a human ribosome to 27 MB for an HIV-1 capsid. In the ribosome case, 8 MB is 64 Mbits, so with a transfer rate of 8 Mbit/s it would take 8 s to download all files, whereas at 1 Mbit/s it would take about a minute.

Occasionally, fitted models do not overlay well with their parent map. In some cases, this is because a model has only partial occupancy or is more flexible than the rest of the assembly, *e.g.*, EMD-1063/1ry1 (Halic et al., 2004) and EMD-5275/3j0g (Zhang et al., 2011). However, there are also cases where the lack of overlap is due to annotation issues (*e.g*., a model may be flagged as related to a map but not have been fitted to it) or to mismatches in the coordinate frames of the map and the models. The visual analysis pages have facilitated detection of several such instances, which have subsequently been remediated by EMDataBank or wwPDB curation staff. The volume viewer lists all models that are flagged as having been fitted to an EMDB volume. By default, only models that are at least 40% inside the surface at the recommended contour level are shown in the 3D viewer; other models are only displayed if activated by the user.

The user can interact with the volume viewer applet through an HTML/JavaScript interface. The user may apply clipping to a slab perpendicular to the viewing direction, rotate, translate, and zoom the map, switch fitted models on/off, change the colour and contour level of the map, centre on specific residues, *etc*.

## 3 Slice viewer

The volume viewer described above works best with maps that yield a well-defined surface when displayed at a certain contour level. This is typically the case for single-particle reconstructions and sub-tomogram averages, but usually not for tomographic reconstructions. Moreover, down-sampling can have a deleterious effect on the level of detail that can be visualised. Therefore, a web-based slice viewer has been developed that allows users to interactively view sections of a tomographic reconstruction. Instead of showing a 3D representation of the whole volume, the slice viewer displays one 2D image (*Z* plane) at a time, with an option to animate through the entire stack of planes, Fig. 1b. Constraints imposed by the web browser are at least as serious as in the case of the volume browser because tomograms can be very large. However, rather than down-sampling the tomographic reconstruction, we use a client–server approach where only one plane is sent to the client at a time. The slice viewer makes a best guess about the density range to use for good contrast, but this range can be changed by the user.

Open Microscopy Environment Remote Objects (OMERO) is a client–server system for managing and visualising images and associated data, originally designed for optical microscopy (Allan et al., 2012). OMERO has been adapted for other applications such as genotype analysis, and is also used by image repositories such as *The Cell: An Image Library* (http://www.cellimagelibrary.org/; Orloff et al., 2013) and the *Journal of Cell Biology DataViewer* (http://jcb-dataviewer.rupress.org/; Hill, 2008). In the slice viewer, OMERO was modified to make it compatible with EMDB data. For the slice viewer this included removing features not relevant for 3DEM (*e.g.*, support for colour channels), and adding new features such as details about the size of the current view and comprehensive help text.

The slice viewer is presently available for tomograms only, at URLs of the form http://pdbe.org/EMD-NNNN/slice, where “EMD-NNNN” is an EMDB accession number, for instance EMD-1906.

## 4 Visual analysis

**Fig.3:**
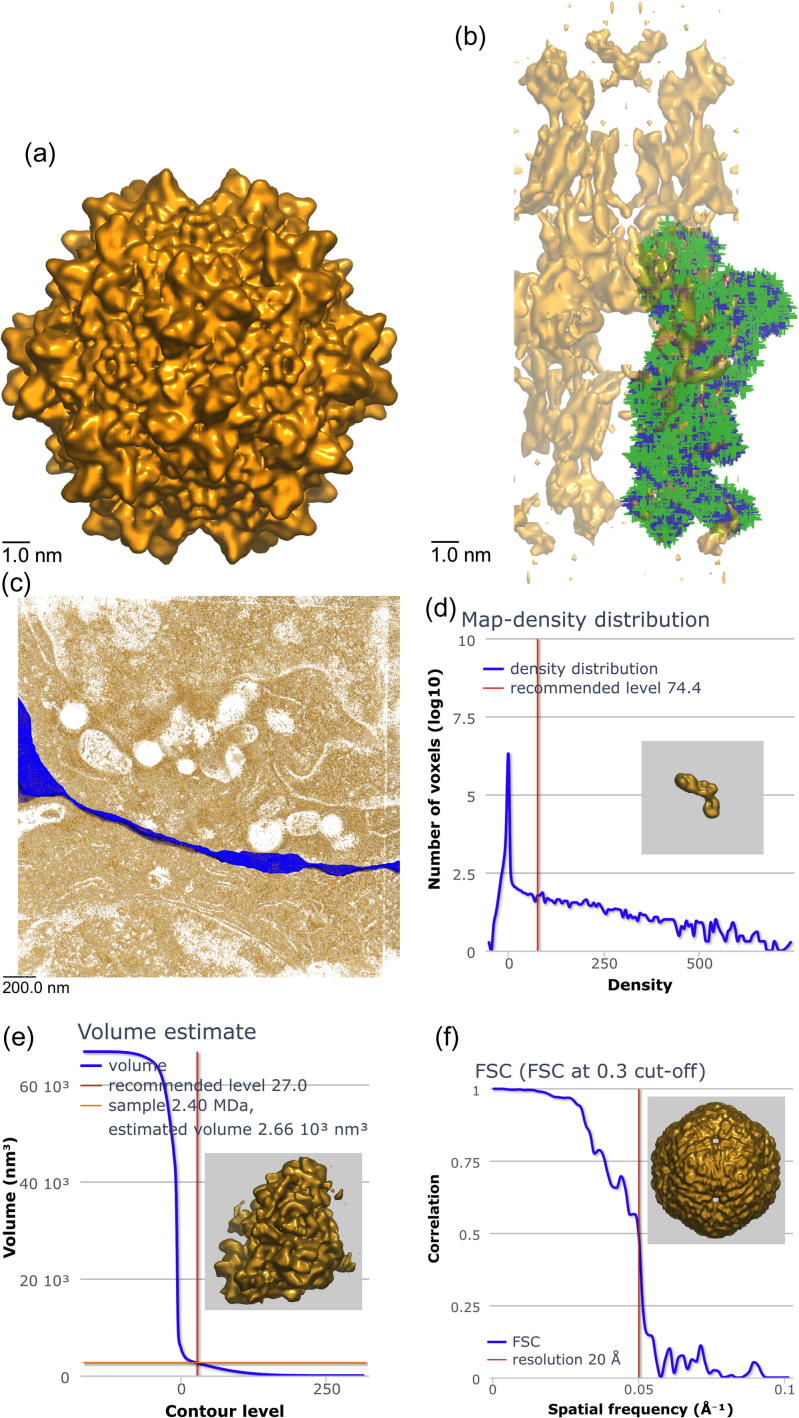

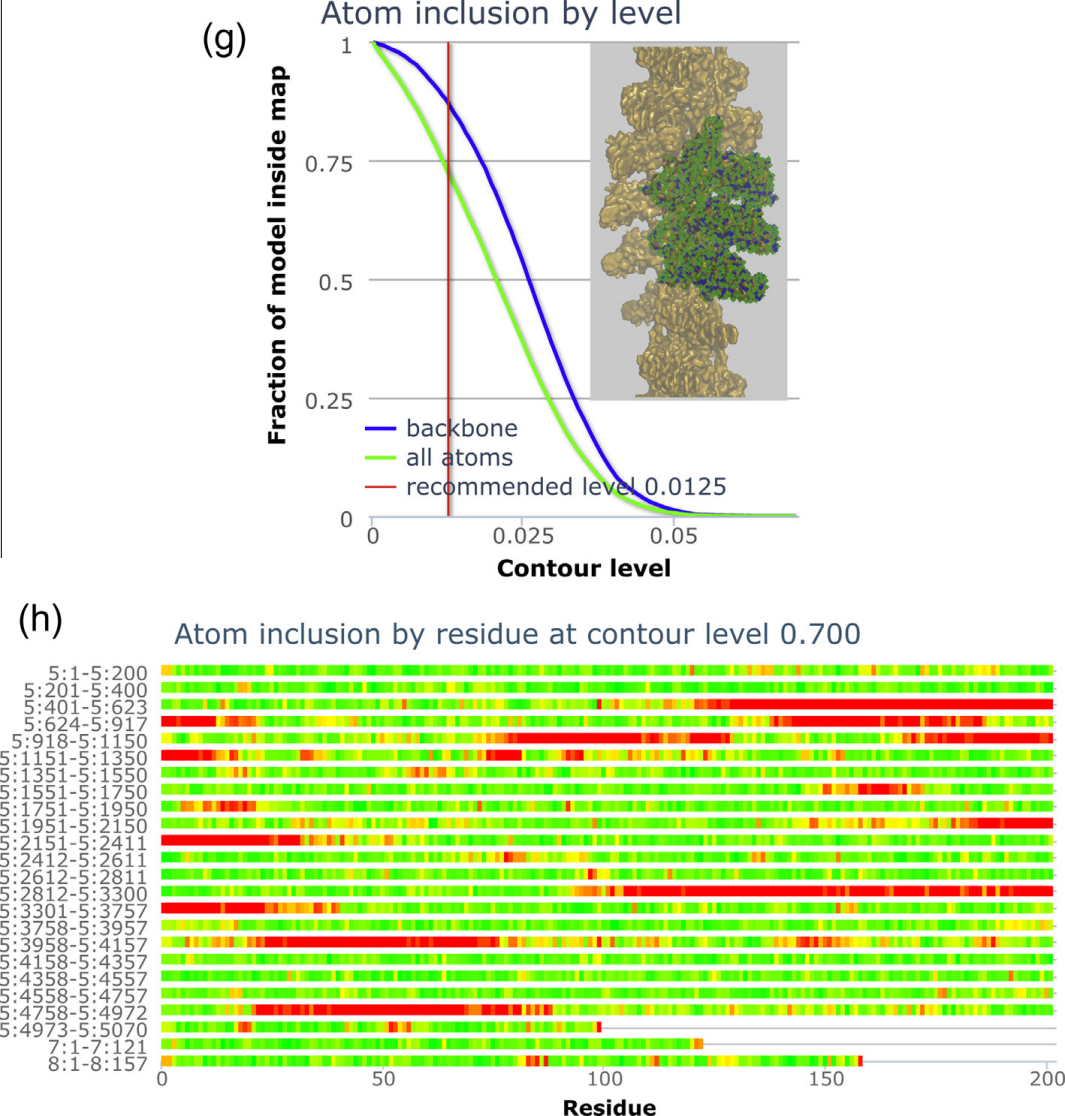
Visual analysis page components (see main text for further details). (a) View along the *X*-axis of EMD-1836, adeno-associated virus type 1 (Gerlach et al., 2011). The visual analysis pages also include views along the *Y* and *Z* axes. (b) View along *X*-axis of EMD-1831 with fitted PDB entry 2xzb, pig gastric H,K-ATPase (Abe et al., 2011). Backbone atoms are shown in blue, side-chain atoms in green. (c) View along the *Z*-axis of EMD-1273, the immunological synapse between a cytotoxic T lymphocyte and a target cell (Stinchcombe et al., 2006). The blue object is a segmentation of the synaptic cleft provided by the depositors. (d) Map-density plot for EMD-1916, *T. thermophilus* ribosome recycling factor (Yokoyama et al., 2012). The spike at zero density indicates that the volume has been masked prior to deposition. (e) Enclosed map-volume plot for EMD-2168, *S. cerevisiae* 60S ribosomal subunit in complex with Rei1 (Greber et al., 2012). The volume of the map is plotted as a function of contour level. The graph and the two lines intersect in one point, suggesting that the contour level results in a map whose volume is consistent with the value estimated from the molecular weight of the specimen. This is one of the better examples in EMDB and more commonly the lines do not intersect in one point. (f) FSC curve for EMD-5357, bacteriophage phi6 procapsid (Nemecek et al., 2011). (g) Fractional atom inclusion as a function of contour level for PDB entry 4a7f fitted to EMD-1987, F-actin-myo1E-tropomyosin complex (Behrmann et al., 2012). Separate curves are shown for trace/backbone atoms only (blue) and all atoms (green). (h) Atom inclusion by residue for PDB entry 3j3f fitted to EMD-5592, human 60S rRNA (Anger et al., 2013). The colour-ramping indicates the extent to which the atoms of a residue are inside or outside the map, with green indicating 100% inside, and red 100% outside the map. (For interpretation of the references to colour in this figure legend, the reader is referred to the web version of this article.)

There is a growing awareness in the community that the results of 3DEM experiments cannot simply be taken at face value and that the development of tools and procedures for validation is essential, as exemplified by the establishment of the Electron Microscopy Validation Task Force (EM VTC; Henderson et al., 2012). Such tools will need to address the validation of maps, models and the quality of the fit of models to maps. We have developed visual analysis pages that provide information to support basic validation of EMDB entries. The visual analysis pages for individual entries are accessible at URLs of the form http://pdbe.org/EMD-NNNN/analysis, where “EMD-NNNN” is an EMDB accession number, for instance EMD-2132. A visual analysis page may contain any or all of the following components (depending on what information is available in the archives), see Fig. 3:

• map-related information:
(a) orthogonal projections
(b) either orthogonal surface views of the map for non-tomogram entries or orthogonal central sections of the map for tomogram entries;
(c) histogram showing the map-density distribution;
(d) plot of the enclosed map volume as a function of contour level;
(e) Fourier Shell Correlation (FSC) plot.
• for each fitted model:
(a) orthogonal surface views of the map with the fitted model overlaid;
(b) plot of the fraction of fitted model atoms inside the map volume as a function of contour level;
(c) a graph of the degree of atom inclusion inside the map volume per residue at the recommended contour level.
• for each segmentation (referred to as “masks” in EMDB):
(a) set of 3 orthogonal surface views of the map with that segmentation overlaid.

All images, graphs and plots are static and prepared in advance because of the high memory and CPU demands for some large entries (up to 25 GB of RAM and >1 CPU hour). All graphs and plots are rendered with the Highcharts JavaScript library (http://www.highcharts.com/), while the map, model and mask images are generated with our customised version of OAV (see above) and Bsoft (Heymann et al., 2008). Examples of the various components of a visual analysis page are shown in Fig. 3 and discussed below.

### 4.1 Orthogonal projections, central sections and surface views

We use the modified version of OAV for the generation of surface-view images and Bsoft (Heymann et al., 2008) for orthogonal projections and central sections. Deposited maps are used without compressing the density range or down-sampling. All images are subsequently rescaled to 300 × 300 and 900 × 900 pixels for presentation on the visual analysis pages. The 300 × 300 pixel images are shown on the page and double-clicking them brings up the corresponding 900 × 900 pixel image, Fig. 3a.

For entries with fitted models an additional set of images is generated for each fitted model in turn with the map surface rendered at 50% transparency, viewed along the *X*, *Y* and *Z* axes, Fig. 3b. If the model contains at least the backbone atoms, then the backbone is shown in blue and any other atoms in green. Backbone atoms are defined as: N, C, CO and CA for amino acids; C3′, C4′, C5′, O3′, O5′ and P for nucleotides; and the first atom encountered for any other residue or compound. For more coarse-grained models the trace atoms (see above) are shown in blue.

If any masks (segmentations) have been deposited for an entry, a set of orthogonal surface projections, with the map surface rendered at 50% transparency, is displayed for each mask, Fig. 3c. For some entries, deposited masks contain sub-tomograms without any frame information, or sub-tomogram averages constructed from multiple sub-tomograms. In those cases, the joint map/mask images will not provide any useful insight.

### 4.2 Map-density distribution

The map-density distribution is shown as a histogram with 128 bins and a logarithmic representation of the voxel count for each bin, Fig. 3d. A sharp spike in the histogram near a density value of zero usually indicates that the volume has been masked. The map-density distribution histograms often have a wider peak around zero due to background scattering and noise outside the sample object. While masking is common practice in 3DEM reconstruction, the EMDB data model does not presently allow this information to be captured. The map-density distribution histogram allows users to assess the extent of masking, which may affect their interpretation of the map.

The contour level used in the generation of the images is marked in the graph. If there is a recommended contour level for an entry, be it provided by the author or by EMDB staff, this value is used. If there is no recommended level, the average density plus one standard deviation is used instead. These latter values obviously depend strongly on the padding of the map and any masking that may have been applied.

### 4.3 Enclosed map volume

One of the plots on the visual analysis pages shows how the enclosed volume of the map varies as a function of contour level. The contour level used to generate the images (see above) is indicated by a vertical line and the intersection of this line and the curve yields the volume of the enclosed surface at that contour level. If the authors recommended a contour level, then the corresponding volume is used to interpret the map. A simple sanity check is to compare this volume estimate with the value that corresponds to the estimated molecular weight of the specimen.

Estimating volume from a reported molecular weight is not straightforward. The reported molecular weight may refer exactly to the reconstructed system, but in some cases it refers to a larger or smaller entity. For instance the weight provided for fibres may be that of the repeating unit or of the entire fibre, and for viruses the weight for the repeating unit or the whole assembly may have been provided, even if the map contains only a half, a quarter or one eighth of the virus particle. Improvements have been made to the EMDB deposition system and data model to try and minimise this kind of ambiguity in the future. Another problem is that the mass density of macromolecular complexes varies considerably (Kantardjieff and Rupp, 2003), with estimates ranging from 1.2 g/cm^3^ for some proteins to almost 2 g/cm^3^ for nucleic acids with CsCl salts. Other factors that affect the appropriate choice of mass density value include the resolution of the map, the use of negative staining and the presence of solvation layers. A mass density value of 1.5 g/cm^3^ is used here to obtain rough volume estimates. It is tacitly assumed that the density is insensitive to freeze plunging and is uniform across samples.

The map volume is plotted as a function of contour level (in 128 bins). The volume is calculated simply by multiplying the number of voxels above the threshold and the voxel volume. More sophisticated volume-calculation methods (Connolly, 1985; Voronoi, 1908) could give more precise values, but this would not be warranted by the accuracy of the estimates. A vertical line marks the contour level used for the images. If the molecular weight is available, it is converted to a volume estimate and indicated by a horizontal line. Ideally, the curve and the two lines should intersect in one point, Fig. 3e; however, there are many entries (and explanations) where this is not the case.

### 4.4 FSC curve

Fourier-Shell Correlation (FSC) (Harauz and van Heel, 1986) is the most commonly used method to estimate the resolution of single-particle EM maps. It entails Fourier transforming two maps and plotting the correlation between the Fourier transforms as a function of spatial frequency. The shape of the FSC curve depends on a number of factors such as any imposed symmetry and masking, and whether or not the two 3D reconstructions that are compared were processed from a common reference. Moreover, different threshold criteria are in use in the community for estimating the resolution from the FSC curve. For validation purposes it is useful to show the full FSC curve rather than a single resolution value. FSC data can be deposited to EMDB, but at present it is available for only 13 entries. Where available, the FSC curve is shown on the visual analysis page, Fig. 3f. Otherwise, only the author-provided resolution and the method used to estimate it are listed. Note that resolution is not a mandatory data item for tomograms in EMDB.

### 4.5 Atom inclusion

If there are fitted PDB models for an EMDB entry, an atom-inclusion graph is generated and displayed, showing the fraction of atoms that are inside the map surface as a function of the contour level, Fig. 3g. Models fitted in 3DEM volumes may either contain trace atoms (see above), backbone atoms, all heavy atoms, or a mixture of these. Side chains are usually poorly resolved at resolutions worse than ∼4 Å (Branden and Tooze, 1991), although density averaging of multiple copies of a molecule or assembly can be used to improve the signal-to-noise ratio and thereby improve the definition of side-chain details. However, these limitations on the resolvability of side chains do not always deter authors from building all-atom models from scratch and depositing them, even at considerably lower resolution than 4 Å. Atom-inclusion information is shown separately for all atoms (if deposited) and for reduced models (backbone or trace atoms).

Atom-inclusion information is also displayed on a per-residue basis with reference to the contour level used for the images, Fig. 3h. Each residue is represented by a small coloured bar, which is red if a residue lies entirely outside the map volume, green if it lies entirely inside, or an intermediate colour if it lies partially inside the map volume. Residues are shown in segments of up to 200, in the same order as they appear in the model. All available atoms are included in the calculations. For models with only trace atoms, the result is binary (either inside or outside).

The map-density distribution, enclosed volume and atom-inclusion graphs are all shown for the entire dynamic range of density values. The enclosed volume and atom inclusion calculations work even in the few cases where the recommended contour level is negative. Finally, the user can interactively zoom the graphs.

## 5 Weekly updates

EMDB operates on a weekly release cycle, synchronised with that of the PDB. New entries are released at midnight UTC on Wednesdays. All images and graphs for new or modified entries are generated ahead of time using an automated procedure that is run on 25 nodes of the EMBL-EBI compute cluster. The most resource-intensive part of this process is the preparation of the surface images, as this is the only step that requires that the surface of the full map be rendered. The time to process an entry varies significantly; most take less than a minute, but more complex entries with many associated models and masks can take a few hours.

## 6 Concluding remarks

The main motivation for developing the tools described here was to make 3DEM data more easily accessible and interpretable, not only to the 3DEM community, but also to a wider audience of non-specialist users. For this reason, the focus has been on developing browser-based visualisation tools that do not require any special software to be installed on the client side. Some compromises have had to be made to work within the constraints imposed by the browsers, such as the down-sampling of large maps for the volume viewer. Should a user wish to examine a map in more detail, it can of course be downloaded from the EMDB archive and studied using a range of dedicated tools such as Chimera and PyMol.

The slice viewer enables interactive browser-based viewing of tomographic reconstructions. Viewing tomograms becomes much more useful and instructive if biologically annotated segmentations can be overlaid. Together with members of the 3DEM community, we are developing a segmentation-file format (http://pdbe.org/emschema, Patwardhan et al. (2012)) that will build on existing formats and will help bring considerably more segmentation data into the archive. A key feature of the new format will be that segments can be linked to biological ontologies such as GO (http://geneontology.org; The Gene Ontology Consortium, 2000) and archives such as UniProt (http://uniprot.org; The UniProt Consortium, 2012) using common identifiers. Once implemented, adopted and supported by the slice viewer, this will turn the slice viewer into a launch pad for other relevant biological and possibly medical resources.

With regards to both the volume viewer and the slice viewer, several challenges and opportunities are presented by technological developments. Several security issues have been reported for Java applets in the past few years; the US National Vulnerability Database lists almost 200 hits when searching for Java 1.7 vulnerabilities (NIST’s National Vulnerability Database; http://web.nvd.nist.gov/view/vuln/search-results?adv_search=true&cves=on&cpe_vendor=oracle&cpe_product=cpe%3A%2F%3Aoracle%3Ajre&cpe_version=cpe%3A%2F%3Aoracle%3Ajre%3A1.7.0), (Michael Mimoso’s threatpost blog; http://threatpost.com/javas-losing-security-legacy). Security concerns have led some browser suppliers to disable Java by default. The instructions a user has to follow to enable Java are often unclear and vary with the browser and operating system. Furthermore, new operating systems such as iOS and Android do not support Java applets at all. If current trends persist, the use of Java applets (such as OAV) in web-based applications will cease to be viable. We are therefore evaluating alternative technologies such as WebGL. In terms of technical advances, although we have seen large increases in computer memory, there have not been concomitant increases in the memory allocated to browser applications. We also do not anticipate any substantial increases in average transfer speeds in the next few years. Therefore, the steps that have been taken to reduce memory usage and data transfers in our viewers are expected to be relevant and necessary for some time to come.

The visual analysis pages begin to address some of the EM VTF recommendations and challenges (Henderson et al., 2012). While the field is still under rapid development, we have begun by targeting the “low-hanging fruit”: visual sanity checks and basic validation information that can be readily compiled from the EMDB archive. While these are only small steps in improving the interpretation of EM volumes, they constitute a significant advance in this field in the way simple visualisation, analysis and validation tools are combined to allow users to assess the contents and reliability of deposited 3DEM data and models. For instance, the combination of the map-density, enclosed volume and atom-inclusion graphs make it possible to assess if the recommended contour level is appropriate, or if a different choice would be more appropriate. The effect of such a choice on the appearance of a map (and any models fitted to it) can subsequently be assessed interactively by changing the contour level in the volume viewer. A PDBe webinar on “Exploring EMDB” (http://www.youtube.com/ProteinDataBank) includes an example of how the visual analysis pages, in combination with the volume viewer, can be used to analyse a map in detail, and to assess the fit of models to a map.

In implementing the tools described here, we have become aware of some of the limitations of the metadata presently archived and of the current EMDB data model, including the ambiguity of the specified molecular weight, and the lack of metadata describing how the FSC calculations were carried out. These issues are all being addressed. Also, we will continue to consult with the 3DEM community on how to expand and improve the visual analysis pages. As the field develops, new insights are gained, and a consensus emerges about how to analyse and validate 3DEM data, we will update the tools and develop new ones where this is opportune.

## Competing financial interests

J.R. Swedlow is affiliated with Glencoe Software, Inc., an open-source US-based commercial company that contributes to OMERO.
